# Mapping the evidence on racialized mothers’ postpartum mental health in Canada: A scoping review protocol

**DOI:** 10.1371/journal.pone.0352132

**Published:** 2026-08-26

**Authors:** Saima Hirani, Savitha Balachandran, Rubee Dev, Kayoung Lee, Michelle Carter

**Affiliations:** 1 University of British Columbia, Faculty of Applied Sciences, School of Nursing, Vancouver, British Columbia, Canada; 2 St. Paul’s Hospital, Vancouver, British Columbia, Canada; National Institute of Mental Health and Neurosciences: National Institute of Mental Health and Neuro Sciences, INDIA

## Abstract

The postpartum period is a time of increased vulnerability to mental health challenges due to physiological, emotional, and social changes, with nearly one-quarter of Canadian mothers reporting symptoms of postpartum depression or anxiety. These challenges may be amplified for racialized mothers, who may encounter systemic barriers such as stigma, discrimination, and limited access to culturally appropriate care. This scoping review aims to map and synthesize the current body of literature on postpartum mental health of racialized mothers living in Canada. This review will be conducted in accordance with the Preferred Reporting Items for Systematic Reviews and Meta-analyses extension for Scoping Reviews (PRISMA-ScR). Two search phases: databases (CINAHL, MEDLINE, PsycINFO and Cochrane Library) and a grey literature search will be used to identify literature. This review will be guided by Arksey and O’Malley’s framework. All studies conducted in Canada and published in English, relevant to postpartum mental health of racialized mothers will be included. The grey literature will focus on programs and interventions for racialized postpartum mothers. We will screen the literature against the inclusion criteria by two independent reviewers and included studies will be presented in a flow diagram in accordance with the PRISMA-ScR guidelines. Data will be extracted and synthesized according to relevant themes or concepts that emerge from the literature and will be presented in diagrammatic or tabular formats. This will be the first scoping review to identify and synthesize evidence on the postpartum mental health of racialized mothers living in Canada. Our results will inform the development of culturally responsive interventions that address the specific needs of this population.

## Introduction

The postpartum period, typically one year after childbirth [1], is widely recognized as a time of heightened vulnerability to mental health stressors, as new mothers undergo profound physiological changes, emotional fluctuations, and shifts in social roles and responsibilities. These adjustments, combined with factors such as sleep deprivation, hormonal changes, and the demands of infant care can significantly impact maternal mental well-being [2,3]. A study published in 2019 found that nearly one-quarter of mothers who recently gave birth in Canada reported experiencing symptoms consistent with postpartum depression or an anxiety disorder, underscoring the widespread nature of mental health challenges for new mothers [4]. For mothers who identify as racialized, which is defined as individuals who are non-Caucasian in race, non-white in color and non-indigenous [5], these challenges are often complex. Such individuals may face an interplay of barriers, including cultural/familial stigma surrounding mental health, limited availability of culturally or linguistically appropriate interventions, discrimination within healthcare systems, and a lack of representation among healthcare providers [6,7]. These barriers not only hinder help-seeking behaviors but also contribute to delayed diagnosis and inadequate treatment. In 2020, it was found that up to 23% of Canadians belonged to a racialized group, and this figure is continuously rising, indicating that a significant portion of the population may be experiencing unique challenges when accessing postpartum mental health care [8].

This scoping review aims to explore the available literature on the postpartum mental health of racialized mothers living in Canada. Previous studies have reviewed similar concepts in the literature, though most have explored the phenomena solely in migrant or refugee mothers [9,10]. While a portion of racialized mothers may identify as immigrants or refugees, there is also a significant portion born in Canada, who experience unique challenges in accessing care. Furthermore, while there are several studies looking at these phenomena internationally [11–13], our study will be limited to a Canadian population, to explore care and outcomes within our specific healthcare system and create actionable resources that will benefit members of the Canadian public directly.

## Methods

This protocol was developed in accordance with the Preferred Reporting Items for Systematic Reviews and Meta-Analyses Protocols (PRISMA-P) checklist [14]. S1 File provides the PRISMA-P checklist for this protocol. Our review will follow the five-stage scoping review framework developed by Arksey and O’Malley: (i) identifying a research question, (ii) locate appropriate articles, (iii) inclusion criteria, (iv) data organization, and (v) data synthesis and presentation [15].

### i. Identifying a research question

Identifying a research question allows the researcher to focus on the specific objectives they aim to achieve in their review [15]. This scoping reviewing will address the following research question: What is known about the postpartum mental health of racialized mothers living in Canada? This population was chosen to identify the unique challenges faced by racialized mothers and to observe the care services and practices available in Canada.

### ii. Locate appropriate articles

#### Search strategies for the identification of studies.

The search strategy was developed in consultation with an expert health Sciences librarian at the University of British Columbia. For research studies, an initial search was conducted in Cumulative Index to Nursing and Allied Health Literature (CINAHL via EBSCOHost), followed by multiple refinements to optimize the strategy for capturing the most relevant literature (Table 1). This refined strategy was subsequently adapted for use in MEDLINE (via EBSCOHost) (Table 2), PsycINFO (via EBSCO) (Table 3) and Cochrane Library (Table 4) for appropriate vocabulary to ensure comprehensive coverage across databases. To ensure a comprehensive understanding of the topic, grey literature sources including Canada Commons, ProQuest and Government of Canada websites, will be used to obtain relevant documents surrounding programs and initiatives relating to our review question. The search terms Postpartum” AND “Mental Health” AND “Racialized” AND “Canada” will be used to obtain relevant grey literature within these sources.

**Table 1:** CINAHL Search Strategy.

**Population:** (MH “Postnatal Period”) OR (MH “Postpartum Nursing”) OR (MH “Postnatal Care”) OR (MH “Lactation”) OR (MH “Puerperium”) or (MH “Perinatal Period”) OR XB (“after birth*” or “after childbirth*” or “after pregnan*” or lactat* or perinatal or puerperium or postpartum or postnatal)
**AND**
**Focus:** (MH “Mental Health”) OR (MH “Emotions + ”) OR (MH “Mental Health Services + ”) OR (MH “Mental Disorders + ”) OR XB (anxiet* or anxious or depress* or emotion* or fear* or “mental* disorder*” or “mental* health*” or “mental illness*” or “postpartum blues” or ppa or ppd or “psychological distress” or psychos?s or stress* or worries or worry)
**AND**
**Context:** Canad* or “British Columbia” or “Colombie Britannique” or Alberta* or Saskatchewan or Manitoba* or Ontario or Quebec or (“New Brunswick” not “New Jersey”) or “Nouveau Brunswick” or “Nova Scotia” or “Nouvelle Ecosse” or “Prince Edward Island” or Newfoundland or Labrador or Nunavut or NWT or “Northwest Territories” or Yukon or Nunavik or Inuvialuit or Abbotsford or Airdrie or Ajax or Aurora or Barrie or Belleville or Blainville or Brampton or Brantford or Brossard or Burlington or Burnaby or Caledon or Calgary or Cambridge or “Cape Breton” or Chatham or Kent or Chilliwack or Clarington or Coquitlam or Drummondville or Edmonton or “Fort McMurray” or Fredericton or Gatineau or Granby or “Grande Prairie” or Sudbury or Guelph or “Halton Hills” or Iqaluit or Inuvik or Kamloops or “Kawartha Lakes” or Kelowna or Kingston or Kitchener or Langley or Laval or Lethbridge or Levis or Longueuil or “Maple Ridge” or Markham or “Medicine Hat” or Milton or Mirabel or Mississauga or Moncton or Montreal or Nanaimo or “New Westminster” or Newmarket or “Niagara Falls” or “Norfolk County” or “North Bay” or “North Vancouver” or Oakville or Oshawa or Ottawa or Peterborough or Pickering or “Port Coquitlam” or “Prince George” or “Quebec City” or “Red Deer” or Regina or Repentigny or (Richmond not Virginia) or “Richmond Hill” or Saanich or Saguenay or “Saint John” or “Saint-Hyacinthe” or “Saint-Jean-sur-Richelieu” or “Saint-Jerome” or Sarnia or Saskatoon or “Sault Ste Marie” or Sherbrooke or “St Albert” or “St Catharines” or “St John's” or “Strathcona County” or Surrey or Terrebonne or “Thunder Bay” or Toronto or “Trois-Rivieres” or Vancouver or Vaughan or ((Halifax or Hamilton or London or Victoria or Waterloo or Welland or Whitby or Windsor) not (UK or “United Kingdom” or Britain or England or Australia)) or Whitehorse or Winnipeg or “Wood Buffalo” or Yellowknife [16]
**AND**
**Participant characteristics:** (MH “People of Color + ”) OR (MH “Minority Groups + ”) OR (MH “Ethnic Groups”) OR (MH “Africans + ”) OR (MH “Black Persons + ”) OR (MH “Australasians + ”) OR (MH “Asians + ”) OR (MH “Arabs + ”) OR (MH “Caribbean Persons + ”) OR (MH “Central Americans”) OR (MH “Creoles”) OR (MH “Jews + ”) OR (MH “Kurds + ”) OR (MH “Mediterranean Persons + ”) OR (MH “Middle Eastern Persons + ”) OR (MH “Rohingya Persons”) OR (MH “Roma”) OR (MH “South Americans + ”) OR (MH “Immigrants + ”) OR (MH “Refugees”) OR (MH “Asian Canadians + ”) OR (MH “Black Canadians”) OR XB (“people of colo#r” or “person* of colo#r” or minorit* or ethnic* or racial* or race or immigra* or migrant* or refugee*)

**Table 2:** MEDLINE Search Strategy.

**Population:** (MH “Peripartum Period”) OR (MH “Postnatal Care”) OR (MH “Postpartum Period + ”) OR XB (“after birth*” or “after childbirth*” or “after pregnan*” or lactat* or perinatal or puerperium or postpartum or postnatal)
**AND**
**Focus:** (MH “Emotions + ”) OR (MH “Mental Disorders + ”) OR (MH “Mental Health”) OR (MH “Mental Health Services + ”) OR XB (anxiet* or anxious or depress* or emotion* or fear* or “mental* disorder*” or “mental* health*” or “mental illness*” or “postpartum blues” or ppa or ppd or “psychological distress” or psychos?s or stress* or worries or worry)
**AND**
**Context:** Canad* or “British Columbia” or “Colombie Britannique” or Alberta* or Saskatchewan or Manitoba* or Ontario or Quebec or (“New Brunswick” not “New Jersey”) or “Nouveau Brunswick” or “Nova Scotia” or “Nouvelle Ecosse” or “Prince Edward Island” or Newfoundland or Labrador or Nunavut or NWT or “Northwest Territories” or Yukon or Nunavik or Inuvialuit or Abbotsford or Airdrie or Ajax or Aurora or Barrie or Belleville or Blainville or Brampton or Brantford or Brossard or Burlington or Burnaby or Caledon or Calgary or Cambridge or “Cape Breton” or Chatham or Kent or Chilliwack or Clarington or Coquitlam or Drummondville or Edmonton or “Fort McMurray” or Fredericton or Gatineau or Granby or “Grande Prairie” or Sudbury or Guelph or “Halton Hills” or Iqaluit or Inuvik or Kamloops or “Kawartha Lakes” or Kelowna or Kingston or Kitchener or Langley or Laval or Lethbridge or Levis or Longueuil or “Maple Ridge” or Markham or “Medicine Hat” or Milton or Mirabel or Mississauga or Moncton or Montreal or Nanaimo or “New Westminster” or Newmarket or “Niagara Falls” or “Norfolk County” or “North Bay” or “North Vancouver” or Oakville or Oshawa or Ottawa or Peterborough or Pickering or “Port Coquitlam” or “Prince George” or “Quebec City” or “Red Deer” or Regina or Repentigny or (Richmond not Virginia) or “Richmond Hill” or Saanich or Saguenay or “Saint John” or “Saint-Hyacinthe” or “Saint-Jean-sur-Richelieu” or “Saint-Jerome” or Sarnia or Saskatoon or “Sault Ste Marie” or Sherbrooke or “St Albert” or “St Catharines” or “St John's” or “Strathcona County” or Surrey or Terrebonne or “Thunder Bay” or Toronto or “Trois-Rivieres” or Vancouver or Vaughan or ((Halifax or Hamilton or London or Victoria or Waterloo or Welland or Whitby or Windsor) not (UK or “United Kingdom” or Britain or England or Australia)) or Whitehorse or Winnipeg or “Wood Buffalo” or Yellowknife [16]
**AND**
**Participant characteristics:** (MH “Asian People + ”) OR (MH “Black People + ”) OR (MH “Emigrants and Immigrants + ”) OR (MH “Ethnic and Racial Minorities”) OR (MH “Middle Eastern and North Africans + ”) OR (MH “Refugees”) OR XB (arab* or black or china or chinese or ethnic* or filipin* or immigra* or japan* or korea* or “latin america*” or migrant* or minorit* or “people of colo#r” or “person* of colo#r” or race or racial* or refugee* or “south asia*” or “south#east asia*” or “west asia*”)

**Table 3:** PsycINFO Search Strategy.

**Population:** (DE “Lactation”) OR (DE “Perinatal Period”) OR (DE “Postnatal Care”) OR (DE “Postnatal Period”) OR XB (“after birth*” or “after childbirth*” or “after pregnan*” or lactat* or perinatal or puerperium or postpartum or postnatal)
**AND**
**Focus:** (DE “Emotions + ”) OR (DE “Emotional Health”) OR (DE “Mental Disorders + ”) OR (DE “Mental Health”) OR (DE “Mental Health Services + ”) OR (DE “Parental Mental Health”) OR XB (anxiet* or anxious or depress* or emotion* or fear* or “mental* disorder*” or “mental* health*” or “mental illness*” or “postpartum blues” or ppa or ppd or “psychological distress” or psychos?s or stress* or worries or worry)
**AND**
**Context:** Canad* or “British Columbia” or “Colombie Britannique” or Alberta* or Saskatchewan or Manitoba* or Ontario or Quebec or (“New Brunswick” not “New Jersey”) or “Nouveau Brunswick” or “Nova Scotia” or “Nouvelle Ecosse” or “Prince Edward Island” or Newfoundland or Labrador or Nunavut or NWT or “Northwest Territories” or Yukon or Nunavik or Inuvialuit or Abbotsford or Airdrie or Ajax or Aurora or Barrie or Belleville or Blainville or Brampton or Brantford or Brossard or Burlington or Burnaby or Caledon or Calgary or Cambridge or “Cape Breton” or Chatham or Kent or Chilliwack or Clarington or Coquitlam or Drummondville or Edmonton or “Fort McMurray” or Fredericton or Gatineau or Granby or “Grande Prairie” or Sudbury or Guelph or “Halton Hills” or Iqaluit or Inuvik or Kamloops or “Kawartha Lakes” or Kelowna or Kingston or Kitchener or Langley or Laval or Lethbridge or Levis or Longueuil or “Maple Ridge” or Markham or “Medicine Hat” or Milton or Mirabel or Mississauga or Moncton or Montreal or Nanaimo or “New Westminster” or Newmarket or “Niagara Falls” or “Norfolk County” or “North Bay” or “North Vancouver” or Oakville or Oshawa or Ottawa or Peterborough or Pickering or “Port Coquitlam” or “Prince George” or “Quebec City” or “Red Deer” or Regina or Repentigny or (Richmond not Virginia) or “Richmond Hill” or Saanich or Saguenay or “Saint John” or “Saint-Hyacinthe” or “Saint-Jean-sur-Richelieu” or “Saint-Jerome” or Sarnia or Saskatoon or “Sault Ste Marie” or Sherbrooke or “St Albert” or “St Catharines” or “St John's” or “Strathcona County” or Surrey or Terrebonne or “Thunder Bay” or Toronto or “Trois-Rivieres” or Vancouver or Vaughan or ((Halifax or Hamilton or London or Victoria or Waterloo or Welland or Whitby or Windsor) not (UK or “United Kingdom” or Britain or England or Australia)) or Whitehorse or Winnipeg or “Wood Buffalo” or Yellowknife [16]
**AND**
**Participant characteristics:** ((DE “Asians + ”) OR (DE “Black People”)OR (DE “Latinos/Latinas”) OR (DE “Minority Groups”) OR (DE “People of Color”) OR (DE “Racial, Ethnic, and Cultural Measures”) OR (DE “Racial and Ethnic Groups”) OR XB (arab* or black or china or chinese or ethnic* or filipin* or immigra* or japan* or korea* or “latin america*” or migrant* or minorit* or “people of colo#r” or “person* of colo#r” or race or racial* or refugee* or “south asia*” or “south#east asia*” or “west asia*”)

**Table 4:** Cochrane Search Strategy.

**Population:** “after birth” or “after childbirth” or “after pregnan” or lactat* or perinatal or puerperium or postpartum or postnatal
**AND**
**Focus:** anxiet* or anxious or depress* or emotion* or fear* or “mental disorder” or “mental health” or “mental illness” or “postpartum blues” or ppa or ppd or “psychological distress” or psychoses or psychosis or stress* or worries or worry
**AND**
**Context:** Canad* or “British Columbia” or “Colombie Britannique” or Alberta* or Saskatchewan or Manitoba* or Ontario or Quebec or (“New Brunswick” not “New Jersey”) or “Nouveau Brunswick” or “Nova Scotia” or “Nouvelle Ecosse” or “Prince Edward Island” or Newfoundland or Labrador or Nunavut or NWT or “Northwest Territories” or Yukon or Nunavik or Inuvialuit or Abbotsford or Airdrie or Ajax or Aurora or Barrie or Belleville or Blainville or Brampton or Brantford or Brossard or Burlington or Burnaby or Caledon or Calgary or Cambridge or “Cape Breton” or Chatham or Kent or Chilliwack or Clarington or Coquitlam or Drummondville or Edmonton or “Fort McMurray” or Fredericton or Gatineau or Granby or “Grande Prairie” or Sudbury or Guelph or “Halton Hills” or Iqaluit or Inuvik or Kamloops or “Kawartha Lakes” or Kelowna or Kingston or Kitchener or Langley or Laval or Lethbridge or Levis or Longueuil or “Maple Ridge” or Markham or “Medicine Hat” or Milton or Mirabel or Mississauga or Moncton or Montreal or Nanaimo or “New Westminster” or Newmarket or “Niagara Falls” or “Norfolk County” or “North Bay” or “North Vancouver” or Oakville or Oshawa or Ottawa or Peterborough or Pickering or “Port Coquitlam” or “Prince George” or “Quebec City” or “Red Deer” or Regina or Repentigny or (Richmond not Virginia) or “Richmond Hill” or Saanich or Saguenay or “Saint John” or “Saint-Hyacinthe” or “Saint-Jean-sur-Richelieu” or “Saint-Jerome” or Sarnia or Saskatoon or “Sault Ste Marie” or Sherbrooke or “St Albert” or “St Catharines” or “St John's” or “Strathcona County” or Surrey or Terrebonne or “Thunder Bay” or Toronto or “Trois-Rivieres” or Vancouver or Vaughan or ((Halifax or Hamilton or London or Victoria or Waterloo or Welland or Whitby or Windsor) not (UK or “United Kingdom” or Britain or England or Australia)) or Whitehorse or Winnipeg or “Wood Buffalo” or Yellowknife [16]
**AND**
**Participant characteristics:** (arab* or black or china or chinese or ethnic* or filipin* or immigra* or japan* or korea* or “latin america” or migrant* or minorit* or “people of color” or “people of colour” or “person of colour” or “person of color” or race or racial* or refugee* or “south asia” or “south east asia” or “west asia”)

The search was conducted in June 2025. The scoping review was planned to be conducted between **August 2025 and February 2026**. This protocol was submitted in July 2025. Due to the duration of peer review, the review has since been completed; no methodological changes were made.

### iii. Inclusion and exclusions criteria

#### Types of studies.

All study designs will be considered for inclusion in this review, except for abstract only from studies and conference proceedings and study protocols. Relevant grey literature, including policy briefs, recommendations and dissertations, will also be considered. This review will include only literature published in English, with no restrictions on publication date.

#### Participants.

The population of interest consists of racialized postpartum mothers within one year of childbirth, and living in Canada. The term “racialized” in this study refers to persons, excluding Indigenous peoples, who are non-Caucasian in race or not white in colour [4]. This definition ensures the review captures the diverse experiences of racialized mothers in the Canadian postpartum context. For mixed population studies, we will only include studies that specifically define a sub-analysis for racialized populations

Concept Included studies and grey literature will focus on the postpartum mental health and well-being of racialized women living in Canada. This review aims to examine research that explores factors influencing mental health during the postpartum period, and the services and programs available in Canada to address mental health concerns or promote mental health well-being of racialized women. It may also investigate the facilitators and barriers these mothers face in accessing appropriate mental health care and support. Additionally, this review will consider studies that explore mothers’ perspectives on their postpartum experiences, including their views on the quality, accessibility, and cultural relevance of the care they received.

#### Context.

This review will be restricted to the Canadian context. Any relevant literature surrounding this study population in a given setting or context will be included. Research conducted in clinics, hospitals, schools, home visits and local community health organizations will be considered for inclusion.

#### Study selection.

Studies retrieved from the previously mentioned databases will be imported into the Covidence, an online reference management platform [17] where duplicate records will be identified and removed. Title and abstract screening will be conducted independently by a minimum of two reviewers to assess relevance based on the predefined inclusion criteria. Studies deemed irrelevant at this stage will be excluded. Full-text articles of potentially eligible studies will then be assessed independently by at least two reviewers to determine final inclusion. Any discrepancies or disagreements arising during either the title/abstract or full-text screening phases will be resolved through discussion or adjudication by a third independent reviewer until consensus is achieved. The study selection process will be documented and presented in accordance with the PRISMA extension for Scoping Reviews (PRISMA-ScR) guidelines [18], using a flow diagram to illustrate the number of records identified, screened, included, and excluded, along with detailed reasons for exclusion to ensure transparency. The retrieved grey literature will be manually imported into an Excel sheet and screened by two independent reviewers to maintain methodological rigor.

### iv. Data organization

#### Data extraction.

Relevant data will be extracted using a data extraction form within the Covidence platform. Extraction will be conducted independently by two reviewers to ensure accuracy and consistency. Any discrepancies or disagreements that arise during the data extraction process will be resolved through discussion, with the involvement of a third reviewer as necessary to achieve consensus. In cases where study full texts are unavailable for data extraction, attempts to contact study authors by email will be made. Data containing study authors, title, publication year, province of study, study design, objective, context/setting, and participant characteristics (including age and ethnicities), and key findings relevant to this review’s question will be extracted. For grey literature, key information, including publication type, organization, intervention or program details, population focus, and outcomes will be charted.

### v. Data synthesis and presentation

The number of studies included at each stage of this review will be presented in a flowchart format, per the PRISMA-ScR guidelines [18]. The research team will collaboratively evaluate the included studies’ aims, designs, and objectives to best synthesize the data to answer our research question. Extracted data from both the peer reviewed studies and grey literature will be synthesized through categorization into prominent themes and concepts that emerge from the literature. The findings from this review will be presented in a narrative format and key findings will be visualized in diagrammatic or tabular formats.

## Discussion

This review aims to evaluate the existing literature about the postpartum mental health of racialized mothers living in Canada, by synthesizing evidence from both peer-reviewed studies and grey literature.

Promoting positive postpartum well-being is essential, as it significantly influences the long-term physical, emotional, and psychological health of both mothers and their children. These impacts extend well beyond the immediate postpartum period, shaping developmental outcomes and overall family health. In the context of Canada’s richly diverse population, it is especially important to consider the unique experiences of racialized mothers, whose postpartum mental health may be shaped by a complex interplay of personal, familial, cultural, and systemic factors. For many in these communities, mental health is a stigmatized issue, and barriers such as cultural misunderstandings, limited access to culturally appropriate care, and structural inequities can hinder timely recognition of postpartum mental health issues and effective support. Given these challenges, this scoping review is positioned to critically examine and synthesize existing evidence on the postpartum mental health experiences and needs of racialized mothers in Canada. Despite its comprehensive search strategy and adherence to validated scoping review frameworks, this proposed review may be limited by the exclusion of studies published in languages other than English, resulting from the lack of language skills within the research team to assess studies in non-English languages.

By identifying current evidence, uncovering knowledge gaps, and exploring the barriers faced by these populations, the review aims to provide a comprehensive basis for future research and support the development of culturally responsive interventions that address the specific needs of racialized postpartum mothers.

## Supporting information

S1 File PRISMA-P checklist. (DOCX)
